# Investigation of *PDCD1* Gene Polymorphisms and Haplotypes in COVID‐19 Severity and Outcome in a Brazilian Population

**DOI:** 10.1002/jmv.71090

**Published:** 2026-07-30

**Authors:** Sarah Lott Moretto, Pedro Luis Candido de Souza Cassela, Guilherme Lerner Trigo, Marcell Alysson Batisti Lozovoy, Zuleica Naomi Tano, Andrea Name Colado Simão, Karen Brajão de Oliveira

**Affiliations:** ^1^ Department of Immunology, Parasitology and General Pathology, Biological Sciences Center State University of Londrina Londrina Paraná Brazil; ^2^ Department of Applied Pathology, Clinical and Toxicological Analysis State University of Londrina Londrina Paraná Brazil; ^3^ Department of Clinical Medicine State University of Londrina Londrina Paraná Brazil

**Keywords:** PD‐1.3, PD‐1.5, PD‐1.6, PD‐1.9, SARS‐CoV‐2 infection

## Abstract

COVID‐19 severity and survival are influenced by the host immune response to SARS‐CoV‐2. Programmed cell death 1 (PD‐1), a key immune checkpoint, regulates T‐cell activation and antiviral immune balance. Since genetic variability can modulate these responses, we investigated whether the *PDCD1* polymorphisms rs11568821 C > T, rs2227982 G > A, rs2227981 G > A and rs10204525 C > T are associated with COVID‐19 severity and mortality in a Brazilian cohort. A total of 366 COVID‐19 patients (165 mild, 72 moderate, and 129 severe cases) were genotyped for the four PDCD1 SNPs, and their haplotype structures were estimated. Significant differences were observed in the allele frequencies of rs11568821, and in genotypes and allele frequencies of the exonic rs2227982, among mild, moderate, and severe cases. Multinomial logistic regression identified associations between rs2227982 (dominant and overdominant models) and moderate COVID‐19, and between the rs2227981 AA genotype (genotypic and recessive models) and severe COVID‐19. The rs10204525 polymorphism (CT genotype under genotypic dominant and overdominant models) also presented an association with severe COVID‐19. However, none of these associations remained independent. Haplotype analysis identified five major haplotypes with significantly different frequencies among the groups (*p* = 0.02); however, no association was found between the haplotypes and disease severity or outcome. This is the first study to evaluate SNP rs2227982 in COVID‐19 patients, and the first to evaluate the four aforementioned SNPs in a Brazilian population. Overall, our findings suggest that these polymorphisms, although involved in COVID‐19 immunopathogenesis, are not suitable biomarkers for predicting COVID‐19 severity or clinical outcomes.

**Abbreviations**
ACE2: angiotensin converting enzyme 2
APCs: antigen presenting cells
CI: confidence interval
CMV: cytomegalovirus
COPD: chronic obstructive pulmonary disease
CTLs: CD8+ cytotoxic T lymphocytes
DCs: dendritic cells
HBV: Hepatitis B virus
HCV: Hepatitis C virus
IL‐6: interleukin‐6
LD: linkage disequilibrium
OR: odds ratio
PD‐1: programmed cell death protein 1
SARS: severe acute respiratory syndrome
SNP: single nucleotide polymorphisms
sPD‐1: soluble PD‐1
TMPRSS2: transmembrane serine protease 2
TNF‐α: tumor necrosis factor α
WHO: World Health Organization

## 1 Introduction

The severe acute respiratory syndrome coronavirus 2 (SARS‐CoV‐2), the etiological agent of coronavirus disease 2019 (COVID‐19), emerged in late 2019 and rapidly spread worldwide, leading to the COVID‐19 pandemic [1, 2]. SARS‐CoV‐2 belongs to the *Coronaviridae* family and the *Betacoronavirus* genus and is an enveloped, positive‐sense single‐stranded RNA virus. Similar to other highly pathogenic human coronaviruses, SARS‐CoV‐2 can cause severe respiratory disease and death, particularly in susceptible individuals, although severe outcomes may also occur in previously healthy individuals [2, 3].

SARS‐CoV‐2 infection is commonly accompanied by a dysregulated immune response characterized by hyperinflammation, commonly referred to as “cytokine storm.” Studies have reported increased neutrophil counts in infected individuals, along with elevated production of pro‐inflammatory cytokines, including interleukin‐6 (IL‐6) and tumor necrosis factor‐alpha (TNF‐α) [4, 5]. These immune alterations have been consistently associated with increased disease severity and mortality [6].

Susceptibility to moderate and severe COVID‐19 is influenced by multiple factors, including the host‐adaptive immune response [7]. One of the key regulators of adaptive immunity is programmed cell death protein 1 (PD‐1), an immune checkpoint receptor highly expressed on the surface of immune cells, including CD8^+^ cytotoxic T lymphocytes (CTLs), antigen‐presenting cells (APCs), and B lymphocytes. PD‐1 plays a crucial role in regulating immune responses and has been implicated in several pathological conditions, including cancer, autoimmune diseases and viral infections [8].

PD‐1 exerts its immunoregulatory functions through binding to its ligands, programmed cell death ligand 1 (PD‐L1) and programmed cell death ligand 2 (PD‐L2), which mediate immune cell activation and peripheral tolerance, thereby preventing inappropriate immune responses against self‐antigens [9, 10, 11]. However, dysregulation of the PD‐1/PD‐L1 axis has been associated with the development of cancer and autoimmune diseases [12, 13]. In the context of viral infections, the PD‐1/PD‐L1 axis has also been recognized as a key regulator of immune responses and plays an important role in the pathogenesis of diseases such as AIDS and hepatitis B [14].

Dysregulation of the PD‐1/PD‐L1 axis has been reported in patients with severe COVID‐19, with PD‐L1 overexpression observed in dendritic cells (DCs), monocytes, eosinophils, and basophils [15, 16, 17]. Furthermore, patients with severe COVID‐19 exhibit T cells with increased PD‐1 expression and an exhausted phenotype [18, 19], possibly due to the infection of these cells via the ACE2 receptor [20, 21].

Genetic variants in the *PDCD1* gene may influence PD‐1 function by disrupting regulatory element binding sites or by altering the amino acid sequence and, consequently, the structure and function of the encoded protein. To the best of our knowledge, only two studies have investigated the association between *PDCD1* gene single nucleotide polymorphisms (SNPs) and COVID‐19 outcomes [22, 23]. However, neither study found significant associations between *PDCD1* genotypes and disease severity or patient survival.

In the present study, we investigated four *PDCD1* SNPs and their corresponding haplotypes, PD‐1.3 C > T (rs11568821; intron 4), PD‐1.9 G > A (rs2227982, exon 5, missense mutation, ala > val), PD‐1.5 G > A (rs2227981, exon 5, synonymous mutation) and PD‐1.6 C > T (rs10204525, 3′ UTR region), in a cohort of Brazilian patients infected with SARS‐CoV‐2. To the best of our knowledge, this is the first study to evaluate these *PDCD1* variants in a Brazilian population and the first to investigate the PD‐1.9 polymorphism in patients with COVID‐19.

## 2 Materials and Methods

### 2.1 Ethical Aspects and Sample Characterization

This study has been approved by the Human Ethics Committee of the State University of Londrina, Paraná, Brazil (CAAE 36247920.1.0000.5231). A consent term was obtained from all patients after a detailed explanation regarding this study. All participants were recruited from the same geographic region and healthcare setting. Peripheral blood samples (5 mL) were collected with EDTA as an anticoagulant agent in the University Hospital of the State University of Londrina, Londrina, Paraná, Brazil (HU‐UEL), between March and October 2020. The inclusion criteria were: confirmed SARS‐CoV‐2 infection by RT‐PCR, availability of peripheral blood collected before COVID‐19 vaccination, availability of clinical data in medical records, and classification of disease severity according to WHO parameters—described below. The exclusion criteria were: absence of RT‐PCR confirmation, previous COVID‐19 vaccination before sample collection, insufficient DNA quantity or quality for genotyping, lack of clinical information necessary for severity classification, and known familial relationship with another participant.

In total, 366 samples were obtained from patients infected with SARS‐CoV‐2, of which 165 presented mild symptoms, 72 had moderate symptoms, and 129 were considered severe COVID‐19 patients, according to the World Health Organization (WHO) parameters. Mild conditions were defined as patients who presented mild clinical symptoms without evidence of pneumonia or hypoxia. Patients in moderate condition presented signs of nonsevere pneumonia, including cough, fever, dyspnea, and oxygen saturation ≥90% on room air. Severe conditions included patients who developed severe pneumonia characterized by O2 saturation less than 90% in room air or a respiratory rate greater than 30 breaths per minute, in addition to symptoms such as cough, dyspnea, and fever. Critical patients characterized by worsening severe pneumonia and the presence of SARS were included in the severe group. The mild COVID‐19 group was considered the control group for all analyses. Sociodemographic and clinicopathological parameters data, such as comorbidities and symptoms, were provided by HU‐UEL through medical records.

### 2.2 Genomic DNA Extraction

Genomic DNA was obtained from peripheral blood leukocytes using Biopur Mini Spin Plus Kit (Biometrix Diagnostica, Curitiba, Brazil), according to the manufacturer instructions. All samples were quantified by NanoDrop 2000c Spectrophotometer (Thermo Scientific, Wilmington, USA) at a wavelength of 260/280 nm, and the final preparations were stored at −20°C until their use.

### 2.3 *PDCD1* genotyping

Quantitative polymerase chain reaction (qPCR) was performed to genotype the SNPs PD‐1.3 (C__57931290_10), PD‐1.9 (C__57931287_20), PD‐1.5 (C__57931286_20) and PD‐1.6 (C____172862_10) using allele‐specific TaqMan MGB probes according to the manufacturer's protocol (Thermo Fisher Scientific, Wilmington, USA). All assays were performed using 1.1 ng/uL of genomic DNA (final concentration of 2.5 ng) on StepOne equipment (Applied Biosystems, Foster City, CA, USA). Negative controls were used in all assays to confirm that no contamination occurred. The genotype was automatically attributed to each sample measuring the allele‐specific fluorescence in the StepOne Software v2.3 (Applied Biosystems).

### 2.4 Haplotype Inference

The inference of recombination sites between alleles of single‐nucleotide variations of *PDCD1* was performed using the PHASE software version 2.1.1 [24, 25]. Only haplotypes with a frequency higher than 5% were chosen for multinomial logistic regression analysis of COVID‐19 severity and disease outcome.

### 2.5 Linkage Disequilibrium Analysis

The linkage disequilibrium (LD) analysis was performed with Haploview software version 4.2 [26] in order to obtain the LD plot and D′, r2, and LOD scores to evaluate LD strength between the four before‐mentioned *PDCD1* SNPs.

### 2.6 Statistical Analysis

First, we conducted the Chi square test (Pearsons's chi square test or Fisher's exact test) to evaluate demographic characteristics, comorbidities, most common symptoms and PD‐1 genotype frequencies between groups. All parameters (demographic, comorbidities, and symptoms) that were significantly associated with the severity of the disease or its outcome were included as confounding variables in the multinomial logistic regression analysis. Then, we estimated the odds ratio (OR) and the 95% confidence interval (95% CI) using multinomial logistic regression analyses to investigate associations between polymorphisms and haplotype structures with severity and disease outcome. All statistical analyses were performed with SPSS 22.0 version (SPSS Inc., Chicago, USA) and were two‐tailed, with a 5% significance level.

## 3 Results

The age of overall COVID‐19 patients ranged from 19 to 95, and the mean age was 55 years (±19.3). For the mild COVID‐19 patients (control group), the age ranged from 21 to 90, and the mean age was 44 (±16.8) years. The moderate and severe COVID‐19 patients ranged from 30 to 90 and 19 to 95 years, respectively, and the mean age was 59 (±18) and 67 (±15.9) years, respectively.

The demographic characteristics, comorbidity parameters, and most common symptoms are shown in Supporting Information S1: Tables 1, 2, and 3, respectively. The association analysis between demographic characteristics and the severity of COVID‐19 (Supporting Information S1: Table 1) indicated that biological sex, age, and survival outcome were associated with the severity of the disease (*p* = 0.001, *p* < 0.001, and *p* < 0.001, respectively).

The comorbidities heart disease (*p* < 0.001), chronic kidney disease (*p* < 0.001), type 2 diabetes (*p* < 0.001), and chronic obstructive pulmonary disease (COPD) (*p* < 0.001) were associated with the severity of the disease, but only the first three were associated with the survival outcome (*p* = 0.001, *p* = 0.039 and *p* = 0.001, respectively) (Supporting Information S1: Table 2).

Among the symptoms studied, all were significantly associated with the severity of COVID‐19 (*p* < 0.001 for all symptoms and *p* = 0.024 for nausea/vomiting), but only fever (*p* = 0.009), loss of smell (*p* = 0.003), loss of taste (*p* = 0.008), difficulty breathing (*p* = 0.05) and SARS occurrence (*p* < 0.001) were associated with the outcome (Supporting Information S1: Table 3).

The genotyping of the evaluated SNPs was not successful for all samples. The variants PD‐1.3 and PD‐1.6 were 100% genotyped, while genotyping for PD‐1.9 and PD‐1.5 was successful in 96.7% (*n* = 354) and 98.1% (*n* = 359) of the samples. All genotypes were in Hardy–Weinberg equilibrium (*p* > 0.05). Linkage disequilibrium analysis between the four SNPs analyzed demonstrated that PD‐1.3 and PD‐1.9 presented strong LD but weak correlation (D′ = 1.0, LOD > 9.0, and *r* ^2^ > 0.2) (Supporting Information S1: Figure 1), suggesting a possible haplotype block. The best pair was PD‐1.9 and PD‐1.6, which presented the strongest LD (D1 = 1.0, LOD > 30, and *r* ^2^=0.316). However, other pairs showed significant LD, but weak correlation. All LD data can be seen in Supporting Information S1: Table 4.

The genotype distribution and allele frequencies are shown in Table 1. The allele distribution of PD‐1.3 varied significantly between groups (*p* = 0.049) but not the genotype distribution (*p* = 0.219). The distribution of the PD‐1.9 genotypes (*p* = 0.047) and alleles (*p* = 0.023) was statistically different between groups.

**Table 1:** X2 association test between allelic and genotypic frequencies of PDCD1 polymorphisms on COVID‐19 mild, moderate and severe groups.

SNP ID (location)	Genotype	COVID‐19 severity			*p* value
		Mild [*n* (%)]	Moderate [*n* (%)]	Severe [*n* (%)]	
rs11568821 PD‐1.3^a^ (Intron 4)	CC	126 (76.4)	63 (87.5)	109 (84.5)	0.219^‡^
	CT	36 (21.8)	9 (12.5)	19 (14.7)	
	TT	3 (1.8)	0 (0)	1 (0.8)	
	Total count	165	72	129	
	Allele C	288 (87.3)	135 (93.7)	237 (91.9)	0.049†*
	Allele T	42 (12.7)	9 (6.3)	21 (8.1)	
rs2227982 PD‐1.9^b^ (Exon 5)	GG	138 (86.8)	47 (71.2)	107 (82.9)	0.047^‡^*
	GA	20 (12.6)	17 (25.8)	21 (16.3)	
	AA	1 (0.6)	2 (3)	1 (0.8)	
	Total count	159	66	129	
	Allele G	269 (93.1)	111 (84.1)	235 (91.1)	0.023†*
	Allele A	22 (6.9)	21 (15.9)	23 (8.9)	
rs2227981 PD‐1.5^c^ (Exon 5)	GG	56 (34.1)	18 (25.4)	32 (25.8)	0.137†
	GA	79 (48.2)	38 (53.5)	56 (45.2)	
	AA	29 (17.7)	15 (21.1)	36 (29.0)	
	Total count	164	71	124	
	Allele G	191 (58.2)	74 (52.1)	120 (48.4)	0.058†
	Allele A	137 (41.8)	68 (47.9)	128 (51.6)	
rs10204525 PD‐1.6^d^ (3′ UTR)	CC	107 (64.8)	40 (55.6)	67 (51.9)	0.087‡
	CT	49 (29.7)	25 (34.7)	56 (43.4)	
	TT	9 (5.5)	7 (9.7)	6 (4.7)	
	Total count	165	72	129	
	Allele C	263 (79.7)	105 (72.9)	190 (73.6)	0.134†
	Allele T	67 (20.3)	39 (27.1)	68 (26.4)	

*Note:* ^†^Pearson's chi square. ^‡^Fisher's exact test. PD‐1.3 (rs11568821), PD‐1.9 (rs2227982), PD‐1.5 (rs2227981) and PD‐1.6 (rs10204525). *p‐values < 0.05 were considered statistically significant. ^a^X^2^ in HWE for mild group = 0.05, *p* > 0.05; moderate group = 0.32, *p* > 0.05; severe group = 0.03, *p* > 0.05. ^b^X^2^ in HWE for mild group = 0.09, *p* > 0.05; moderate group = 0.09, *p* > 0.05; severe group = 0.0007, *p* > 0.05. ^c^X^2^ in HWE for mild group = 0.015, *p* > 0.05; moderate group = 0.37, *p* > 0.05; severe group = 1.14, *p* > 0.05. ^d^X^2^ in HWE for mild group = 1.12, *p* > 0.05; moderate group = 1.05, *p* > 0.05; severe group = 1.8, *p* > 0.05. Genotyping was not 100% successful in all 366 samples for all four SNPs analyzed.

Then, we proceeded with multinomial logistic regression, analyzing PD‐1 polymorphisms' various hereditary models and severity of COVID‐19, and the patients' survival outcome (Table 2). The analysis showed a correlation between the dominant and overdominant hereditary models of PD‐1.9 and moderate COVID‐19 (OR = 2.657, CI = 1.315–5.367, *p* = 0.006, and OR = 2.411, CI = 1.169–4.973, *p* = 0.017, respectively). We also found an association between PD‐1.5 AA genotype under genotypic model (OR = 2.172, CI = 1.129–4.178, *p* = 0.020) and recessive model and (OR = 1.904, CI = 1.090–3.327, *p* = 0.024) severe COVID‐19. The following hereditary models of PD‐1.6 showed correlation with the severe form of the infection: genotypic for CT genotype (OR = 1.825, CI = 1.118–2.980, *p* = 0.016), dominant (OR = 1.707, CI = 1.066–2.733, *p* = 0.026), and overdominant (OR = 1.816, CI = 1.121–2.942, *p* = 0.015). However, after considering the variables sex, age, heart disease, chronic kidney disease, type 2 diabetes, and COPD (chronic obstructive pulmonary disease) as confounding factors (Table 3), all correlations mentioned above lost significance, indicating that the SNPs are not independent factors for COVID‐19 severity.

**Table 2:** Association analysis of the effect of *PDCD1* polymorphisms on the severity of COVID‐19.

		COVID‐19 severity						
Hereditary model		Mild	Moderate			Severe		
	SNP	*N*	*N*	OR (95% CI)	*p* value	*N*	OR (95% CI)	*p* value
PD‐1.3								
Dominant	CC	126	63	—		109	—	
	CT + TT	39	9	0.462 (0.210–1.012)	0.054	20	0.593 (0.326–1.077)	0.086
Overdominant	CT	36	9	—		19	—	
	CC + TT	129	63	0.512 (0.232–1.128)	0.097	110	0.619 (0.336–1.141)	0.124
PD‐1.9								
Dominant	GG	138	47	—		107	—	
	GA + AA	21	19	2.657 (1.315–5.367)	0.006*	22	1.351 (0.706–2.586)	0.363
Overdominant	GA	20	17	—		21	—	
	GG + AA	139	49	2.411 (1.169–4.973)	0.017*	108	1.351 (0.697–2.620)	0.373
PD‐1.5								
Genotypic	GG^a^	56	18	—		32	—	
	GA^b^	79	38	1.496 (0.776–2.887)	0.229	56	1.241 (0.714–2.157)	0.445
	AA^c^	29	15	1.609 (0.710–3.650)	0.255	36	2.172 (1.129–4.178)	0.020*
Dominant	GG	56	18	—		32	—	
	GA + AA	108	53	1.527 (0.818–2.851)	0.184	92	1.491 (0.890–2.497)	0.129
Overdominant	GA	79	38	—		56	—	
	GG + AA	85	33	1.239 (0.709–2.165)	0.452	68	0.886 (0.555–1.415)	0.612
Recessive	GG + GA	135	56	—		88	—	
	AA	29	15	1.247 (0.621–2.503)	0.535	36	1.904 (1.090–3.327)	0.024*
PD‐1.6								
Genotypic	CC^a^	107	40	—		67	—	
	CT^b^	49	25	1.365 (0.747–2.495)	0.312	56	1.825 (1.118–2.980)	0.016*
	TT^c^	9	7	2.081 (0.726–5.960)	0.172	6	1.065 (0.363–3.126)	0.909
Dominant	CC	107	40	—		67	—	
	CT + TT	58	32	1.476 (0.840–2.594)	0.176	62	1.707 (1.066–2.733)	0.026*
Overdominant	CT	49	25	—		56	—	
	CC + TT	116	47	1.259 (0.699–2.269)	0.443	73	1.816 (1.121–2.942)	0.015*
Recessive	CC + CT	156	65	—		123	—	
	TT	9	7	1.867 (0.667–5.225)	0.235	6	0.846 (0.293–2.440)	0.756

*Note:* Multinomial logistic regression. Control: mild COVID‐19. Case: moderate and severe COVID‐19. PD‐1.3 (rs11568821), PD‐1.9 (rs2227982), PD‐1.5 (rs2227981) and PD‐1.6 (rs10204525). *p‐values < 0.05 were considered statistically significant. Genotyping was not 100% successful in all 366 samples for all four SNPs analyzed.

Abbreviations: CI, confidence interval; OR, odds ratio.

^a^ Reference genotype: CC or GG.

^b^ Codominant model: CCvsCT or GGvsGA.

^c^ Codominant model: CCvsTT or GGvsAA.

**Table 3:** Adjusted model for association analysis of the effect of *PDCD1* polymorphisms on the severity of COVID‐19.

		COVID‐19 severity						
Hereditary model		Mild	Moderate			Severe		
	SNP	*N*	*N*	Adj. OR (95% CI)	*p* value	*N*	Adj. OR (95% CI)	*p* value
PD‐1.3								
Dominant	CC	126	63	—		109	—	
	CT + TT	39	9	0.525 (0.167–1.645)	0.269	20	0.672 (0.2243–2.020)	0.479
Overdominant	CT	129	63	—		110	—	
	CC + TT	36	9	0.652 (0.201–2.107)	0.474	19	0.825 (0.266–2.563)	0.740
PD‐1.9								
Dominant	GG	138	47	—		107	—	
	GA + AA	21	19	2.099 (0.668–6.595)	0.204	22	0.795 (0.232–2.726)	0.715
Overdominant	GA	20	17	—		21	—	
	GG + AA	139	49	1.829 (0.574–5.821)	0.307	108	0.797 (0.231–2.744)	0.719
PD‐1.5								
Genotypic	GG^a^	56	18	—		32	—	
	GA^b^	79	38	1.127 (0.403–3.148)	0.820	56	0.950 (0.335–2.694)	0.923
	AA^c^	29	15	1.675 (0.460–6.100)	0.434	36	2.095 (0.592–7.420)	0.252
Dominant	GG	56	18	—		32	—	
	GA + AA	108	53	1.269 (0.476–3.385)	0.634	92	1.230 (0.460–3.289)	0.680
Overdominant	GA	79	38	—		56	—	
	GG + AA	85	33	0.892 (0.375–2.121)	0.795	68	0.668 (0.279–1.598)	0.364
Recessive	GG + GA	135	56	—		88	—	
	AA	29	15	1.544 (0.518–4.601)	0.436	36	2.167 (0.747–6.289)	0.155
PD‐1.6								
Genotypic	CC^a^	107	40	—		67	—	
	CT^b^	49	25	0.885 (0.356–2.199)	0.792	56	0.969 (0.396–2.367)	0.944
	TT^c^	9	7	1.335 (0.284–6.268)	0.715	6	0.273 (0.039–1.903)	0.190
Dominant	CC	107	40	—		67	—	
	CT + TT	58	32	0.954 (0.404–2.252)	0.914	62	0.845 (0.358–1.994)	0.700
Overdominant	CT	49	25	0.843 (0.350–2.029)	0.704	56	1.093 (0.458–2.605)	0.841
	CC + TT	116	47	—		73	—	
Recessive	CC + CT	156	65	—		123	—	
	TT	9	7	1.410 (0.317–6.271)	0.652	6	0.277 (0.042–1.848)	0.185

*Note:* Adjusted multinomial logistic regression. The variables sex, age, heart disease, chronic kidney disease, diabetes type 2 and COPD were considered confound factors in the adjusted regression model. *p‐values < 0.05 were considered statistically significant. Adj.: adjusted. Control: mild COVID‐19. Case: moderate and severe COVID‐19. PD‐1.3 (rs11568821), PD‐1.9 (rs2227982), PD‐1.5 (rs2227981) and PD‐1.6 (rs10204525). Genotyping was not 100% successful in all 366 samples for all four SNPs analyzed.

Abbreviations: CI: confidence interval; OR, odds ratio.

^a^ Reference genotype: CC or GG.

^b^ Codominant model: CCvsCT or GGvsGA.

^c^ Codominant model: CCvsTT or GGvsAA.

The analysis of PD‐1 polymorphisms' various hereditary models showed no correlation between the genotypes and the patients survival outcomes (Table 4), even after adjusting the model for the confounding factors mentioned in the previous paragraph (Table 5).

**Table 4:** Association analysis of the effect of *PDCD1* polymorphisms on the survival outcome of COVID‐19 patients.

		Survival outcome			
Hereditary model		Survived	Deceased		
	SNP	*N*	*N*	OR (95% CI)	*p* value
PD‐1.3					
Dominant	CC	136	56	—	
	CT + TT	24	12	1.214 (0.568–2.595)	0.616
Overdominant	CT	23	11	—	
	CC + TT	137	57	1.150 (0.526–2.513)	0.727
PD‐1.9					
Dominant	GG	124	53	—	
	GA + AA	30	15	1.170 (0.582–2.352)	0.660
Overdominant	GA	28	14	—	
	GG + AA	126	54	1.167 (0.570–2.388)	0.673
PD‐1.5					
Genotypic	GG^a^	38	18	—	
	GA^b^	78	30	0.812 (0.403–1.637)	0.560
	AA^3^ c	39	17	0.920 (0.414–2.047)	0.838
Dominant	GG	38	18	—	
	GA + AA	117	47	0.848 (0.440–1.633)	0.622
Overdominant	GA	78	30	—	
	GG + AA	77	35	0.846 (0.474–1.512)	0.573
Recessive	GG + GA	116	48	—	
	AA	39	17	1.053 (0.544–2.041)	0.877
PD‐1.6					
Genotypic	CC^a^	88	32	—	
	CT^b^	61	32	1.398 (0.773–2.526)	0.268
	TT^c^	11	5	1.250 (0.403–3.877)	0.699
Dominant	CC	88	32	—	
	CT + TT	72	36	1.375 (0.778–2.429)	0.273
Overdominant	CT	61	31	—	
	CC + TT	99	37	1.360 (0.766–2.414)	0.294
Recessive	CC + CT	149	63	—	
	TT	11	5	1.075 (0.359–3.221)	0.897

*Note:* Multinomial logistic regression. The variables sex, age, heart disease, chronic kidney disease, diabetes type 2 and COPD were considered confound factors in the adjusted regression model. *p‐values < 0.05 were considered statistically significant. Control: survived patients. Case: deceased patients. PD‐1.3 (rs11568821), PD‐1.9 (rs2227982), PD‐1.5 (rs2227981) and PD‐1.6 (rs10204525). Genotyping was not 100% successful in all 366 samples for all four SNPs analyzed.

Abbreviations: CI, confidence interval; OR, odds ratio.

^a^ Reference genotype: CC or GG.

^b^ Genotypic model: CCvsCT or GGvsGA.

^c^ Genotypic model: CCvsTT or GGvsAA.

**Table 5:** Adjusted association analysis of the effect of PDCD1 polymorphisms on the survival outcome of COVID‐19 patients.

		Survival outcome			
Hereditary model		Survived	Deceased		
	SNP	*N*	*N*	Adj. OR (95% CI)	*p* value
PD‐1.3					
Dominant	CC	136	56	—	
	CT + TT	24	12	0.988 (0.434–2.250)	0.976
Overdominant	CT	23	11	—	
	CC + TT	137	57	0.970 (0.416–2.264)	0.944
PD‐1.9					
Dominant	GG	124	53	—	
	GA + AA	30	15	1.245 (0.574–2.701)	0.579
Overdominant	GA	28	14	—	
	GG + AA	126	54	1.268 (0.572–2.807)	0.559
PD‐1.5					
Genotypic	GG^a^	38	18	—	
	GA^b^	78	30	0.775 (0.357–1.683)	0.520
	AA^c^	39	17	0.659 (0.268–1.621)	0.364
Dominant	GG	38	18	—	
	GA + AA	117	47	0.733 (0.355–1.515)	0.402
Overdominant	GA	78	30	—	
	GG + AA	77	35	0.950 (0.498–1.814)	0.877
Recessive	GG + GA	116	48	—	
	AA	39	17	0.777 (0.367–1.645)	0.509
PD‐1.6					
Genotypic	CC^a^	88	32	—	
	CT^b^	61	32	1.318 (0.686–2.533)	0.408
	TT^c^	11	5	0.994 (0.280–3.529)	0.992
Dominant	CC	88	32	—	
	CT + TT	72	36	1.268 (0.674–2.383)	0.461
Overdominant	CT	61	31	—	
	CC + TT	99	37	1.319 (0.702–2.479)	0.390
Recessive	CC + CT	149	63	—	
	TT	11	5	0.868 (0.255–2.952)	0.820

*Note:* Adjusted multinomial logistic regression. The variables sex, age, heart disease, chronic kidney disease, diabetes type 2 and COPD were considered confound factors in the adjusted regression model. *p‐values < 0.05 were considered statistically significant. Adj.: adjusted. Control: survived patients. Case: deceased patients. PD‐1.3 (rs11568821), PD‐1.9 (rs2227982), PD‐1.5 (rs2227981) and PD‐1.6 (rs10204525). Genotyping was not 100% successful in all 366 samples for all four SNPs analyzed.

Abbreviations: CI: confidence interval; OR: odds ratio.

^a^ Reference genotype: CC or GG.

^b^ Genotypic model: CCvsCT or GGvsGA.

^c^ Genotypic model: CCvsTT or GGvsAA.

Afterward, we proceeded to haplotype analysis, that indicated 12 occurring haplotypes on all COVID‐19 groups (Table 6). Permutation analysis showed their frequencies varied significantly between the mild COVID‐19 group and the moderate (*p* < 0.02) and severe (*p* < 0.02) COVID‐19 groups. Multinomial logistic regression analysis indicated a significant association between the TGGC haplotype dominant hereditary model and severe COVID‐19 (Table 7). However, after adjusting the model for confound factors, the association was lost (Table 8). The analysis indicated no association with the survival outcome (Tables 9 and 10), either before or after adjusting the model for confounding factors.

**Table 6:** Haplotype distribution among COVID‐19 groups.

	COVID‐19 severity		
Haplotype	Mild (*n* = 165) [*N* (%)]	Moderate (*n* = 72) [*N* (%)]	Severe (*n* = 129) [*N* (%)]
CGGC	128 (38.71)	49 (34.35)	91 (35.43)
CGAC	93 (28.29)	47 (32.35)	81 (31.23)
CGAT	43 (12.94)	17 (11.48)	42 (16.15)
TGGC	42 (12.67)	9 (6.21)	17 (6.67)
CAGT	20 (6.03)	16 (11.13)	18 (7.04)
CAAT	2 (0.61)	5 (3.4)	5 (1.85)
CGGT	2 (0.68)	1 (1.03)	0 (0.16)
TAGT	0 (0.01)	0 (0.0)	3 (1.14)
TGAC	0 (0.03)	0 (0.03)	1 (0.32)
TGGT	0 (0.02)	0 (0.00)	0 (0.005)
CAGC	0 (0.01)	0 (0.01)	0 (0.001)
CAAC	0 (0.00)	0 (0.01)	0 (0.004)
Total	330 (100.0)	144 (100.0)	258 (100.0)
Permutation *p*‐value	—	0.02*	0.02*

*Note:* Haplotype structures and frequencies were estimated according to the number of allele copies of the SNPs PD‐1.3 (rs11568821), PD‐1.9 (rs2227982), PD‐1.5 (rs2227981) and PD‐1.6 (rs10204525), respectively in this order, using PHASE software version 2.2.1. *Values of *p* < 0.05 were considered statistically significant.

**Table 7:** Association analysis of the effect of *PDCD1* haplotypes on the severity of COVID‐19.

	COVID‐19 severity						
Haplotype model	Mild	Moderate			Severe		
	*N*	*N*	OR (95% CI)	*p* value	*N*	OR (95% CI)	*p* value
CGGC genotypic							
Other/other	65	30	—		59	—	
CGGC/other	73	35	1.039 (0.575–1.876)	0.900	49	0.739 (0.446–1.226)	0.242
CGGC/CGGC	27	7	0.562 (0.220–1.434)	0.228	21	0.857 (0.438–1.675)	0.652
CGGC dominant							
Other/other	65	30	—		59	—	
CGGC/other	100	42	0.910 (0.518–1.598)	0.743	70	0.771 (0.484–1.229)	0.275
CGGC recessive							
Other/other	138	7	—		21	—	
CGGC/CGGC	27	65	0.550 (0.228–1.330)	0.185	108	0.994 (0.533–1.854)	0.984
CGAC genotypic							
Other/other	86	31	—		61	—	
CGAC/other	64	35	1.517 (0.848–2.714)	0.160	56	1.234 (0.759–2.006)	0.397
CGAC/CGAC	15	6	1.110 (0.395–3.114)	0.843	12	1.128 (0.493–2.579)	0.775
CGAC dominant							
Other/other	86	31	—		61	—	
CGAC/other	79	41	1.440 (0.824–2.515)	0.200	68	1.214 (0.765–1.925)	0.411
CGAC recessive							
Other/other	150	66	—		117	—	
CGAT/CGAT	15	6	0.909 (0.338–2.447)	0.850	12	1.026 (0.462–2.275)	0.950
CGAT genotypic							
Other/other	124	55	—		89	—	
CGAT/other1	38	16	0.949 (0.488–1.846)	0.878	38	1.393 (0.824–2.357)	0.216
CGAT/CGAT0	3	1	0.752 (0.076–7.387)	0.806	2	0.929 (0.152–5.674)	0.936
CGAT dominant							
Other/other	124	55	—		89	—	
CGAT/other	41	17	0.935 (0.489–1.788)	0.839	40	1.359 (0.813–2.272)	0.242
CGAT recessive							
Other/other	162	71	—		127	—	
CGAT/CGAT	3	1	0.761 (0.078–7.438)	0.814	2	0.850 (0.140–5.166)	0.860
CAGT dominant							
Other/other	144	15	—		110	—	
CAGT/other	21	57	1.805 (0.869–3.745)	0.113	19	1.184 (0.607–2.311)	0.620
TGGC dominant							
Other/other	125	62	—		111	—	
TGGC/other	40	10	0.504 (0.236–1.074)	0.076	18	0.507 (0.275–0.935)	0.030*

*Note:* Multinomial logistic regression. *p‐values < 0.05 were considered statistically significant. Haplotype structures were estimated according to the number of allele copies of the SNPs PD‐1.3 (rs11568821), PD‐1.9 (rs2227982), PD‐1.5 (rs2227981) and PD‐1.6 (rs10204525), respectively in this order. Genotypic model = all other haplotypes carriers' versus one copy/other carriers and all other haplotypes carriers' versus two copies carriers; Dominant model = all other haplotypes carriers' versus one copy/other carriers; Recessive model = one copy/all other haplotypes carriers' versus two copies carriers. Not all models were applicable to all haplotypes due to their low frequency in the evaluated cohorts.

Abbreviations: CI, confidence interval; OR, odds ratio.

**Table 8:** Adjusted association analysis of the effect of *PDCD1* haplotypes on the severity of COVID‐19.

	COVID‐19 severity						
Haplotype model	Mild	Moderate			Severe		
	*N*	*N*	OR (95% CI)	*p* value	*N*	OR (95% CI)	*p* value
CGGC genotypic							
Other/other	65	30	—		59	—	
CGGC/other	73	35	1.068 (0.416–2.743)	0.891	49	0.854 (0.236–2.234)	0.747
CGGC/CGGC	27	7	0.648 (0.165–2.545)	0.535	21	1.262 (0.357–4.457)	0.718
CGGC dominant							
Other/other	65	30	—		59	—	
CGGC/other	100	42	0.957 (0.397–2.304)	0.921	70	0.960 (0.399–2.311)	0.927
CGGC recessive							
Other/other	138	7	—		21	—	
CGGC/CGGC	27	65	0.625 (172–2.274)	0.476	108	1.358 (0.416–4.432)	0.612
CGAC genotypic							
Other/other	86	31	—		61	—	
CGAC/other	64	35	1.425 (0.582–3.490)	0.438	56	1.134 (0.460–2.799)	0.784
CGAC/CGAC	15	6	2.354 (0.406–13.653)	0.340	12	2.684 (0.497–14.498)	0.251
CGAC dominant							
Other/other	86	31	—		61	—	
CGAC/other	79	41	1.554 (0.660–3.662)	0.313	68	1.334 (0.568–3.130)	0.508
CGAC recessive							
Other/other	150	66	—		117	—	
CGAT/CGAT	15	6	2.004 (0.360–11.158)	0.427	12	2.558 (0.491–13.321)	0.265
CGAT genotypic							
Other/other	124	55	—		89	—	
CGAT/other	38	16	0.617 (0.240–1.590)	0.318	38	0.803 (0.318–2.030)	0.643
CGAT/CGAT	3	1	0.688 (0.035–13.470)	0.806	2	0.533 (0.029–9.844)	0.672
CGAT dominant							
Other/other	124	55	—		89	—	
CGAT/other	41	17	0.619 (0.246–1.556)	0.308	40	0.782 (0.317–1.932)	0.594
CGAT recessive							
Other/other	162	71	—		127	—	
CGAT/CGAT	3	1	0.788 (0.041–15.279)	0.875	2	0.567 (0.031–10.340)	0.702
CAGT dominant							
Other/other	144	15	—		110	—	
CAGT/other	21	57	1.552 (492–4.897)	0.454	19	0.734 (0.211–2.559)	0.628
TGGC dominant							
Other/other	125	62	—		111	—	
TGGC/other	40	10	0.496 (0.165–1.490)	0.212	18	0.548 (0.186–1.617)	0.276

*Note:* Adjusted multinomial logistic regression. The variables sex, age, heart disease, chronic kidney disease, diabetes type 2 and COPD were considered confound factors in the adjusted regression model. *p‐values < 0.05 were considered statistically significant. Haplotype structures were estimated according to the number of allele copies of the SNPs PD‐1.3 (rs11568821), PD‐1.9 (rs2227982), PD‐1.5 (rs2227981) and PD‐1.6 (rs10204525), respectively in this order. Adj.: adjusted. Genotypic model = all other haplotypes carriers' versus one copy/other carriers and all other haplotypes carriers' versus two copies carriers; Dominant model = all other haplotypes carriers' versus one copy/other carriers; Recessive model = one copy/all other haplotypes carriers' versus two copies carriers. Not all models were applicable to all haplotypes due to their low frequency in the evaluated cohorts.

Abbreviations: CI, confidence interval; OR, odds ratio.

**Table 9:** Association analysis of the effect of *PDCD1* haplotypes on the survival outcome of COVID‐19 patients.

	COVID‐19 severity			
Haplotype model	Survived	Deceased		
	*N*	*N*	OR (95% CI)	*p* value
CGGC genotypic				
Other/other	74	30	—	
CGGC/other	63	29	1.135 (0.616–2.092)	0.684
CGGC/CGGC	23	9	0.965 (0.400–2.326)	0.937
CGGC dominant				
Other/other	74	30	—	
CGGC/other	86	38	1.090 (0.616–1.929)	0.767
CGGC recessive				
Other/other	137	59	—	
CGGC/CGGC	23	9	0.909 (0.397–2.081)	0.821
CGAC genotypic				
Other/other	74	4	—	
CGAC/other	70	30	0.933 (0.517–1.682)	0.817
CGAC/CGAC	16	34	0.544 (0.169–1.750)	0.307
CGAC dominant				
Other/other	74	34	—	
CGAC/other	86	34	0.860 (0.488–1.518)	0.604
CGAC recessive				
Other/other	144	64	—	
CGAT/CGAT	16	4	0.563 (0.181–1.749)	0.320
CGAT genotypic				
Other/other	113	47	—	
CGAT/other	44	20	1.093 (0.583–2.049)	0.782
CGAT/CGAT	3	1	0.801 (0.081–7.903)	0.850
CGAT dominant				
Other/other	113	47	—	
CGAT/other	47	21	0.931 (0.502–1.725)	0.820
CGAT recessive				
Other/other	157	67	—	
CGAT/CGAT	3	1	1.280 (0.131–12.531)	0.832
CAGT genotypic				
Other/other	135	55	—	
CAGT/other	23	12	0.781 (0.363–1.678)	0.526
CAGT/CAGT	2	1	0.815 (0.072–9.171)	0.868
CAGT dominant				
Other/other	135	55	—	
CAGT/other	25	13	0.783 (0.374–1.642)	0.518
CAGT recessive				
Other/other	158	67	—	
CAGT/CAGT	2	1	0.848 (0.076–9.513)	0.894
TGGC dominant				
Other/other	134	58	—	
TGGC/other	26	10	1.125 (0.510–2.484)	0.770

*Note:* Multinomial logistic regression. *p‐values < 0.05 were considered statistically significant. Haplotype structures were estimated according to the number of allele copies of the SNPs PD‐1.3 (rs11568821), PD‐1.9 (rs2227982), PD‐1.5 (rs2227981) and PD‐1.6 (rs10204525), respectively in this order. Genotypic model = all other haplotypes carriers' versus one copy/other carriers and all other haplotypes carriers' versus two copies carriers; Dominant model = all other haplotypes carriers' versus one copy/other carriers; Recessive model = one copy/all other haplotypes carriers' versus two copies carriers.

Abbreviations: CI, confidence interval; OR, odds ratio.

**Table 10:** Adjusted association analysis of the effect of *PDCD1* haplotypes on the survival outcome of COVID‐19 patients.

	COVID‐19 severity			
Haplotype model	Survived	Deceased		
	*N*	*N*	Adj. OR (95% CI)	*p* value
CGGC genotypic				
Other/other	74	30	—	
CGGC/other	63	29	1.361 (0.680–2.723)	0.383
CGGC/CGGC	23	9	1.766 (0.659–4.733)	0.258
CGGC dominant				
Other/other	74	30	—	
CGGC/other	86	38	1.451 (0.760–2.769)	0.259
CGGC recessive				
Other/other	137	59	—	
CGGC/CGGC	23	9	1.528 (0.606–3.854)	0.369
CGAC genotypic				
Other/other	74	4	—	
CGAC/other	70	30	0.824 (0.428–1.584)	0.561
CGAC/CGAC	16	34	0.372 (0.101–1.368)	0.137
CGAC dominant				
Other/other	74	34	—	
CGAC/other	86	34	0.733 (0.390–1.376)	0.334
CGAC recessive				
Other/other	144	64	—	
CGAT/CGAT	16	4	0.409 (0.116–1.444)	0.165
CGAT genotypic				
Other/other	113	47	—	
CGAT/other	44	20	0.980 (0.490–1.961)	0.954
CGAT/CGAT	3	1	0.476 (0.040–5.674)	0.577
CGAT dominant				
Other/other	113	47	—	
CGAT/other	47	21	0.939 (0.476–1.853)	0.856
CGAT recessive				
Other/other	157	67	—	
CGAT/CGAT	3	1	0.479 (0.041–5.659)	0.559
CAGT genotypic				
Other/other	135	55	—	
CAGT/other	23	12	1.453 (0.626–3.368)	0.384
CAGT/CAGT	2	1	1.019 (0.079–13.230)	0.988
CAGT dominant				
Other/other	135	55	—	
CAGT/other	25	13	1.409 (0.626–3.175)	0.408
CAGT recessive				
Other/other	158	67	—	
CAGT/CAGT	2	1	0.954 (0.074–12.308)	0.971
TGGC dominant				
Other/other	134	58	—	
TGGC/other	26	10	0.712 (0.304–1.666)	0.433

*Note:* Adjusted multinomial logistic regression. The variables sex, age, heart disease, chronic kidney disease, diabetes type 2 and COPD were considered confound factors in the adjusted regression model. *p‐values < 0.05 were considered statistically significant. Haplotype structures were estimated according to the number of allele copies of the SNPs PD‐1.3 (rs11568821), PD‐1.9 (rs2227982), PD‐1.5 (rs2227981) and PD‐1.6 (rs10204525), respectively in this order. Adj.: adjusted. Genotypic model = all other haplotypes carriers' versus one copy/other carriers and all other haplotypes carriers' versus two copies carriers; Dominant model = all other haplotypes carriers' versus one copy/other carriers; Recessive model = one copy/all other haplotypes carriers' versus two copies carriers.

Abbreviations: CI, confidence interval; OR, odds ratio.

## 4 Discussion

This study is the first comprehensive investigation of the SNPs PD‐1.3 (rs11568821), PD‐1.9 (rs2227982), PD‐1.5 (rs2227981), and PD‐1.6 (rs10204525) in COVID‐19 patients regarding the disease severity and clinical outcomes in a Brazilian population. In our study, we found associations between several demographic characteristics, comorbidities, and symptoms parameters and the severity and survival outcome of COVID‐19 patients. These findings broadly support the work of other studies in this area linking sex, age, heart disease, type 2 diabetes, kidney disease, and chronic obstructive pulmonary disease (COPD) with the severity and survival outcome of COVID‐19, as well described in the review of Zhang et al. [27]. These are well‐established risk factors, significantly influencing the immune system's adequate response and can impair proper anti‐virus response, favoring a worse prognosis, disease progression, and mortality [27].

Interestingly, male patients were more frequently represented in the moderate and severe cases in our study, whereas females predominated in the mild group. As informed before, we observed an association between biological sex and COVID‐19 severity. These findings are in agreement with previous review articles indicating that men are at a higher risk of developing severe forms of COVID‐19 [27, 28, 29, 30]. Differences in COVID‐19 severity between male and female patients may be explained by variations in sex hormones involved in inflammatory responses, differential expression of ACE2 (Angiotensin Converting Enzyme 2) and TMPRSS2 (Transmembrane Serine Protease 2), which are molecules involved in the SARS‐CoV‐2 cell entrance, and lifestyle‐related factors, such as smoking [31]. Altogether, these results suggest that sex represents an important determinant of disease severity.

To adjust for the confounding factors, we used the “common cause” method, which consists of selecting all covariates that are known to be associated with the outcome, in this case, the severity of the disease. This knowledge is based on scientific literature already published. Therefore, we selected age, sex, and the presence of comorbidities (type 2 diabetes, COPD, chronic kidney disease, and heart disease) as covariates to be included in the logistic regression analysis to adjust for confounding.

The present study aimed to elucidate the association between PD‐1 single nucleotide polymorphisms and both COVID‐19 severity and survival outcomes. It is well established that host genetic factors may influence the immune response against SARS‐CoV‐2 and, consequently, the disease survival outcome [32, 33].

We observed that the PD‐1.3 (rs11568821) T allele was significantly associated with the severity of COVID‐19. So far, only one study has been published regarding this SNP and COVID‐19. Damavandi. et al. [23] observed no difference between the genotypes and alleles of the PD‐1.3 among Iranian healthy subjects and COVID‐19 patients. Other studies have assessed this SNP's frequency and influence on the following viral infections: Hepatitis C virus (HCV) infection [34, 35] and Cytomegalovirus (CMV) infection [36] on Iranian, Egyptian and French subjects, respectively, with PD‐1.3 being associated with all three infections. The PD‐1.3 SNP is located at the position +7146 of intron 4 of the *PDCD1* gene and has been associated with cancer and autoimmune diseases before [13, 37]. Kroner et al. [38] showed that this SNP disrupts the RUNX1 transcription factor‐binding site, impairing PD‐1 transcription. One possible explanation for our finding of no association between PD‐1.3 and COVID‐19 severity or disease outcome may be that lower PD‐1 expression could favor a faster viral clearance and better disease outcome due to more active and effective T lymphocytes, as the opposite situation, higher PD‐1 levels, has been linked to inhibition of cell proliferation and decreased effector functions [39].

PD‐1.9 is located on the exon 5 of the *PDCD1* gene, being a non‐synonymous substitution of alanine to valine at codon 215. *PDCD1* exon 5 is known to encode a cytoplasmic domain consisting of two tyrosine motifs that are associated with inhibitory activities. Considering the missense change induced by PD‐1.9, this amino acid shift could influence the structure and function of the *PDCD1* final protein [40]. However, the exact consequence of this polymorphism on final protein structure and function remains to be elucidated. In reviewing the literature, no data were found associating PD‐1.9 (rs2227982) and COVID‐19 disease. We observed that PD‐1.9 genotypes and alleles presented a significant distribution difference among the studied groups (*p* = 0.047, *p* = 0.023, respectively). An association between the dominant (*p* = 0.006) and overdominant (*p* = 0.017) hereditary models and moderate disease was also observed, indicating that A allele carriers have at least twice the chance to have moderate COVID‐19. However, after adjusting the model for confounding variables (sex, age, heart disease, chronic kidney disease, type 2 diabetes, and COPD), the association with COVID‐19 severity was lost. Previous studies have evaluated this SNP regarding Hepatitis B virus (HBV) infection on Chinese population [41, 42] and Vietnamese population [40], and HCV infection on Chinese population [43]. Huang et al. [41] observed a protective factor of GG genotype on HBV infection and Huyen et al. [40], likewise, found that AA genotype was a risk factor for HBV infection, but no correlation between soluble PD‐1 (sPD‐1) was found with this polymorphism. A recent study evaluated sPD‐1 in multiple myeloma patients and PD‐1.9 was linked with higher sPD‐1 expression [44]. Though we haven't measured either PD‐1 expression or its soluble protein form, we hypothesize that A allele could mildly increase PD‐1 expression, which could explain the increased risk of these carriers to develop moderate, but not severe disease, as a higher expression leads to T cell exhaustion and impairs viral clearance. However, this mechanistic interpretation remains speculative. Because the association was no longer significant after adjustment for potential confounding factors, our data do not provide independent statistical evidence that the PD‐1.9 polymorphism influences COVID‐19 severity. Therefore, any proposed biological mechanism should be regarded as a plausible hypothesis rather than a conclusion supported by the present genetic association analysis.

The SNPs PD‐1.5 and PD‐1.6 are located on exon 5 and 3′ UTR, respectively. In this case‐control study, we found associations between the PD‐1.5 AA genotype (*p* = 0.020) under genotypic hereditary model and severe COVID‐19, as well as for the recessive hereditary model (*p* = 0.024). The PD‐1.6 hereditary models associated with severe COVID‐19 were CT genotype under genotypic model (*p* = 0.016), dominant (*p* = 0.026) and overdominant (*p* = 0.015) hereditary models. The literature concerning these SNPs is scarce. Only two studies evaluated them on COVID‐19 patients, both on Iranian patients [22, 23]. Damavandi et al. [23] observed that the PD‐1.5 genotypes significantly differed between control and case groups, but no difference was observed among alleles, and no correlations were observed between this SNP and COVID‐19 severity or survival outcome. The location of PD‐1.5 is the same as PD‐1.9, but the nucleotide shift is considered synonymous, hence, not altering the amino acid reading frame. This could explain the absence of correlation with COVID‐19 severity and mortality, as no structural alteration could result from this SNP, and therefore PD‐1 function remains the same [13]. The analysis of PD‐1.6 conducted by Mirsharif et al. [22] resulted in no association between these genotypes and alleles and COVID‐19 severity of mortality. The location of PD‐1.6 matches miR‐4717 binding site, a microRNA responsible for the *PDCD1* gene expression. The change of base in this exact location has been shown to be disruptive, affecting the association of the microRNA with the DNA sequence, suppressing gene expression [45]. As previously discussed, lower PD‐1 expression could benefit hosts' defense against SARS‐CoV‐2, preventing T cells exhaustion phenotype and improving its effectiveness. Nevertheless, no functional analyses were performed in the present study to confirm this hypothesis, and our genetic findings alone are insufficient to establish a causal relationship between these variants and COVID‐19 severity.

However, studies by Damavandi et al. [23] and Mirsharif et al. [22] considered their control group only asymptomatic with negative RT‐PCR test at the time of sample collection. This approach is a limiting factor for our discussion, since we considered mild COVID‐19 patients as our control group, being a robust control, therefore comparisons should be interpreted with caution. Another limitation is that these studies evaluated susceptibility to COVID‐19 infection without providing information regarding in what conditions these healthy individuals were living during the pandemic (e.g., no contact with infected individuals due to physical isolation; use of personal protective equipment, such as masks) or whether they could have had the asymptomatic form of the disease prior to the investigation. Hence, it is important to be cautious in affirming that these individuals have a lower susceptibility to SARS‐CoV‐2 infection.

Our haplotype analysis identified 12 major haplotypes in the studied population. Five of them (CGGC, CGAC, CGAT, TGGC and CAGT) had frequencies above 5%. Despite having a significant distribution among the COVID‐19 groups, only one haplotype, T_PD‐1.3_G_PD‐1.9_G_PD‐1.5_C_PD‐1.6_ under the dominant hereditary model, was associated with severe COVID‐19. However, after adjusting the model, the association was lost. Damavandi et al. [23] found that the A_PD‐1.3_T_PD‐1.5_ haplotype was significantly more frequent in COVID‐19 patients than in the healthy control group, but this result was inconclusive due to the wide confidence interval range. Therefore, although differences in haplotype frequencies were observed between the clinical groups, these findings cannot be interpreted as evidence of an independent association with COVID‐19 severity. Instead, they should be considered exploratory and hypothesis‐generating until replicated in larger independent populations.

The present study presents some limitations that should be considered. The sample size was moderate for a genetic association study, which may have reduced the power to detect variants with low penetrance or small effect sizes. In addition, although nominal associations were observed, these associations were not maintained after adjustment for established clinical covariates, including age and comorbidities. This finding does not diminish the relevance of the results, but rather reflects the strong influence of clinical factors on COVID‐19 progression and the importance of interpreting genetic associations within this context. Nonetheless, the study also has important strengths. The cohort was well characterized and classified according to standardized WHO criteria, and genotyping was performed using validated probes and rigorous quality control procedures. To our knowledge, this is the first study to evaluate these four *PDCD1* variants in a Brazilian population, and the first to investigate the rs2227982 (PD‐1.9) polymorphism in the context of COVID‐19. Moreover, the admixed genetic background of the Brazilian population provides an opportunity to explore genetic associations in a diverse and still underrepresented group. Therefore, although exploratory, our findings contribute relevant preliminary evidence and may support future studies in larger and multi‐ethnic cohorts.

This type of investigation contributes to expanding scientific knowledge on genetic polymorphisms related to immunomodulatory molecules and their influence on infectious diseases pathogenesis and progression. Taken together, our findings suggest that the evaluated polymorphisms are not suitable as biomarkers for predicting COVID‐19 severity or outcomes in the Brazilian population.

## Author Contributions

**Sarah Lott Moretto:** writing – original draft, methodology, formal analysis, conceptualization, review and editing. **Pedro Luis Candido de Souza Cassela:** writing – review and editing, data curation. **Guilherme Lerner Trigo:** writing – review and editing, methodology. **Marcell Alysson Batisti Lozovoy:** writing – review and editing, methodology. **Zuleica Naomi Tano:** writing – review and editing, methodology, data curation. **Andrea Name Colado Simão:** writing – review and editing, methodology, data curation. **Karen Brajão de Oliveira:** writing – review and editing, supervision, funding acquisition, conceptualization.

## Ethics Statement

This study was reviewed and approved by the Human Ethics Committee of the State University of Londrina, Paraná, Brazil (CAAE 36247920.1.0000.5231).

## Conflicts of Interest

The authors declare no conflicts of interest.

## Supporting information


Supporting File

## Data Availability

The data that support the findings of this study are available on request from the corresponding author. The data are not publicly available due to privacy or ethical restrictions.
