# Supplementary material for: Investigation of PDCD1 Gene Polymorphisms and Haplotypes in COVID‐19 Severity and Outcome in a Brazilian Population
Source: J Med Virol. 2026 Jul 30;98(8):e71090. doi: 10.1002/jmv.71090 (PMC13422133; doi:10.1002/jmv.71090)
Supplement: Supplementary file 1 — Supporting File [file JMV-98-e71090-s001.docx]

**Supplementary material to:**

***PDCD1* gene single nucleotide polymorphisms and haplotypes in COVID-1: severity and outcome in a Brazilian population**

**Microbiological Research**

Moretto, S. L.^1^, Vitiello, G. A. F.^1^, Banin-Hirata, B. K.^2^, Guembarovski, R. L.^3^, Watanabe, M. A. E. ^1^, de Oliveira, K. B.^1^

^1^Department of Immunology, Parasitology and General Pathology, Biological Sciences Center, State University of Londrina, Londrina, PR, Brazil.

^2^Department of Basic Health Sciences, Health Sciences Center, State University of Maringá, Maringá, PR, Brazil

^3^Department of General Biology, Biological Sciences Center, State University of Londrina, Londrina, PR, Brazil.

**Corresponding author:** Karen Brajão de Oliveira; Department of Immunology, Parasitology and General Pathology, Biological Sciences Center, State University of Londrina, Londrina, PR, Brazil; Email: [karen.brajao@gmail.com](mailto:karen.brajao@gmail.com)

**Supplementary table I.** X^2^ association test between demographic characteristics of COVID-19 patients and the severity of the disease.

| **Demographic characteristics** | | **COVID-19 severity** | | |  |
| --- | --- | --- | --- | --- | --- |
|  |  | **Mild** | **Moderate** | **Severe** | ***p*-value** |
|  |  | **N (%)** | **N (%)** | **N (%)** |  |
| **Biological sex** | Female | 99 (60.0) | 28 (38.9) | 52 (40.3) | 0.001^†^* |
|  | Male | 66 (40.0) | 44 (61.1) | 77 (59.7) |  |
| **Ethnicity** | Caucasian | 131 (79.4) | 56 (80.0) | 98 (76.0) | 0.723^†^ |
|  | Non caucasian | 34 (20.6) | 14 (20.0) | 31 (24.0) |  |
| **Age** | 18-39 years | 83 (50.3) | 8 (11.2) | 7 (5.4) | <0.001^†^* |
|  | 40-69 years | 65 (39.4) | 41 (56.9) | 58 (45) |  |
|  | ≥ 70 years | 17 (10.3) | 23 (31.9) | 64 (49.6) |  |
| **Smoker** | No | 162 (98.2) | 66 (91.7) | 125 (96.9) | 0.059^‡^ |
|  | Yes | 3 (1.8) | 6 (8.3) | 4 (3.1) |  |
| **Former smoker** | No | 157 (95.2) | 65 (90.3) | 116 (89.9) | 0.188^†^ |
|  | Yes | 8 (4.8) | 7 (9.7) | 13 (10.1) |  |
| **Survival outcome** | Survived | 34 (100.0) | 65 (97) | 61 (48.4) | <0.001^†^* |
|  | Deceased | 0 (0.0) | 2 (3) | 65 (51.6) |  |

^†^Pearson’s Qui square. ^‡^Fisher’s exact test. *p values <0.05 were considered statistically significant.

**Supplementary table II.** X^2^ association test between comorbidities of COVID-19 patients and the severity and outcome of the disease

|  | | **COVID-19 severity** | | |  | **Survival outcome** | |  |
| --- | --- | --- | --- | --- | --- | --- | --- | --- |
| **Comorbidities** | | **Mild** | **Moderate** | **Severe** | ***p*-value** | **Survived** | **Deceased** | ***p*-value** |
|  |  | **N (%)** | **N (%)** | **N (%)** |  | **N (%)** | **N (%)** |  |
| **Heart disease** | No | 160 (97) | 66 (91.7) | 101 (78.3) | <0.001^†^* | 141 (88.1) | 48 (70.6) | 0.001^†^* |
|  | Yes | 5 (3) | 6 (8.3) | 28 (21.7) |  | 19 (11.9) | 20 (29.4) |  |
| **Chronic kidney disease** | No | 162 (98.2) | 59 (81.9) | 106 (82.2) | <0.001^†^* | 138 (86.3) | 51 (75) | 0.039^†^* |
|  | Yes | 3 (1.8) | 13 (18.1) | 23 (17.8) |  | 22 (13.8) | 17 (25) |  |
| **Type 1 diabetes** | No | 162 (98.2) | 70 (97.2) | 125 (96.9) | 0.747^‡^ | 154 (96.3) | 66 (97.1) | 1.0^‡^ |
|  | Yes | 3 (1.8) | 2 (2.8) | 4 (3.1) |  | 6 (3.8) | 2 (2.9) |  |
| **Type 2 diabetes** | No | 154 (93.3) | 56 (77.8) | 81 (62.8) | <0.001^†^* | 122 (76.3) | 37 (54.4) | 0.001^†^* |
|  | Yes | 11 (6.7) | 16 (22.2) | 48 (37.2) |  | 38 (23.8) | 31 (45.6) |  |
| **COPD** | No | 165 (100) | 68 (94.4) | 124 (96.1) | 0.007^‡^* | 154 (96.3) | 65 (95.6) | 1.0^‡^ |
|  | Yes | 0 (0) | 4 (5.6) | 5 (3.9) |  | 6 (3.8) | 3 (4.4) |  |
| **Hepatopathy** | No | 164 (99.4) | 71 (98.6) | 126 (97.7) | 0.434^‡^ | 157 (98.1) | 66 (97.1) | 0.636^‡^ |
|  | Yes | 1 (0.6) | 1 (1.4) | 3 (2.3) |  | 3 (1.9) | 2 (2.9) |  |
| **HIV infection** | No | 165 (100) | 72 (100) | 127 (98.4) | 0.162^‡^ | 159 (99.4) | 67 (98.5) | 0.508^‡^ |
|  | Yes | 0 (0) | 0 (0) | 2 (1.6) |  | 1 (0.6) | 1 (1.5) |  |
| **Malignant neoplasm** | No | 163 (98.8) | 71 (98.6) | 127 (98.4) | 1.0^‡^ | 158 (98.8) | 66 (97.1) | 0.585^‡^ |
|  | Yes | 2 (1.2) | 1 (1.4) | 2 (1.6) |  | 2 (1.3) | 2 (2.9) |  |
| **Obesity** | No | 134 (81.2) | 58 (80.6) | 103 (79.8) | 0.958^†^ | 133 (83.1) | 55 (80.9) | 0.684^†^ |
|  | Yes | 31 (18.8) | 14 (19.4) | 26 (20.2) |  | 27 (16.9) | 13 (19.1) |  |

^†^Pearson’s qui square. ^‡^Fisher’s exact test. *p values <0.05 were considered statistically significant. COPD: Chronic obstructive pulmonary disease.

**Supplementary table III.** X^2^ association test between most common symptoms of COVID-19 patients and the severity and outcome of the disease.

|  | | **COVID-19 severity** | | |  | **Survival outcome** | |  |  |
| --- | --- | --- | --- | --- | --- | --- | --- | --- | --- |
| **Symptoms** | | **Mild** | **Moderate** | **Severe** | ***p*-value** | **Survived** | **Deceased** | ***p*-value** |  |
|  |  | **N (%)** | **N (%)** | **N (%)** |  | **N (%)** | **N (%)** |  |  |
| **Fever** | No | 110 (66.7) | 23 (31.9) | 61 (47.3) | <0.001* | 64 (40) | 40 (58.8) | 0.009* |  |
|  | Yes | 55 (33.3) | 49 (68.1) | 68 (52.7) |  | 96 (60) | 28 (41.2) |  |  |
| **Headache** | No | 67 (40.6) | 55 (76.4) | 115 (89.1) | <0.001* | 132 (82.5) | 61 (89.7) | 0.167 |  |
|  | Yes | 98 (59.4) | 17 (23.6) | 14 (10.9) |  | 28 (17.5) | 7 (10.3) |  |  |
| **Sore throat** | No | 107 (64.8) | 57 (79.2) | 109 (85.2) | <0.001* | 130 (81.8) | 60 (88.2) | 0.226 |  |
|  | Yes | 58 (35.2) | 15 (20.8) | 19 (14.8) |  | 29 (18.2) | 8 (11.8) |  |  |
| **Diarrhoea** | No | 113 (68.5) | 61 (84.7) | 119 (92.2) | <0.001* | 140 (87.5) | 62 (91.2) | 0.424 |  |
|  | Yes | 52 (31.5) | 11 (15.3) | 10 (7.8) |  | 20 (12.5) | 6 (8.8) |  |  |
| **Nausea/vomiting** | No | 139 (84.2) | 57 (79.2) | 119 (92.2) | 0.024* | 138 (86.3) | 62 (91.2) | 0.3 |  |
|  | Yes | 26 (15.8) | 15 (20.8) | 10 (7.8) |  | 22 (13.8) | 6 (8.8) |  |  |
| **Loss of smell** | No | 76 (46.1) | 58 (80.6) | 125 (96.9) | <0.001* | 141 (88.1) | 68 (100) | 0.003* |  |
|  | Yes | 89 (53.9) | 14 (19.4) | 4 (3.1) |  | 19 (11.9) | 0 (0) |  |  |
| **Loss of taste** | No | 83 (50.3) | 57 (79.2) | 123 (95.3) | <0.001* | 140 (87.5) | 67 (98.5) | 0.008* |  |
|  | Yes | 82 (49.7) | 15 (20.8) | 6 (4.7) |  | 20 (12.5) | 1 (1.5) |  |  |
| **Malaise** | No | 74 (44.8) | 49 (68.1) | 87 (67.4) | <0.001* | 110 (68.8) | 51 (75) | 0.343 |  |
|  | Yes | 91 (55.2) | 23 (31.9) | 42 (32.6) |  | 50 (31.3) | 17 (25) |  |  |
| **Difficulty breathing** | No | 123 (74.5) | 34 (47.2) | 31 (24) | <0.001* | 59 (36.9) | 16 (23.5) | 0.05* |  |
|  | Yes | 42 (25.5) | 38 (52.8) | 98 (76) |  | 101 (63.1) | 52 (76.5) |  |  |
| **SARS** | No | 161 (97.6) | 58 (80.6) | 51 (39.5) | <0.001* | 124 (77.5) | 12 (17.6) | <0.001* |  |
|  | Yes | 4 (2.4) | 14 (19.4) | 78 (60.5) |  | 36 (22.5) | 56 (82.4) |  |  |

Pearson’s qui square. *p values <0.05 were considered statistically significant. SARS: Severe Acute Respiratory Syndrome.

**Supplementary table IV.** LD analysis between *PDCD1* polymorphisms among COVID-19 patients.

| **SNP1** | **SNP2** | **D'** | **LOD** | **r²** |
| --- | --- | --- | --- | --- |
| rs11568821 | rs2227982 | 1.0 | 0.99 | 0.011 |
| rs11568821 | rs2227981 | 0.836 | 6.34 | 0.063 |
| rs11568821 | rs10204525 | 0.867 | 3.02 | 0.026 |
| rs2227982 | rs2227981 | 0.564 | 2.3 | 0.027 |
| rs2227982 | rs10204525 | 1.0 | 30.99 | 0.316 |
| rs2227981 | rs10204525 | 0.428 | 5.29 | 0.065 |

Pairwise linkage disequilibrium (LD) analysis among the SNPs PD-1.3 (rs11568821), PD-1.9 (rs2227982), PD-1.5 (rs2227981) and PD-1.6 (rs10204525) in *PDCD1* gene.


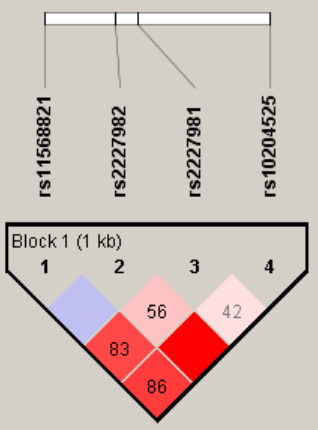


**Supplementary figure I**. Pairwise linkage disequilibrium (LD) among the SNPs PD-1.3 (rs11568821), PD-1.9 (rs2227982), PD-1.5 (rs2227981) and PD-1.6 (rs10204525) in *PDCD1* gene. Numbers in the squares indicate D’ score. LD analysis includes all three groups.
